# Estimating Dynamic Signals From Trial Data With Censored Values

**DOI:** 10.1162/CPSY_a_00003

**Published:** 2017-10-01

**Authors:** Ali Yousefi, Darin D. Dougherty, Emad N. Eskandar, Alik S. Widge, Uri T. Eden

**Affiliations:** 1 Department of Neurological Surgery, Massachusetts General Hospital and Harvard Medical School, Boston, MA; 2 Department of Mathematics and Statistics, Boston University, Boston, MA; 3 Department of Psychiatry, Massachusetts General Hospital and Harvard Medical School, Boston, MA; 4 Picower Institute for Learning & Memory, Massachusetts Institute of Technology, Cambridge, MA

**Keywords:** state-space model, missing data, Bayesian filtering Gaussian approximation, censored data, likelihood function, dynamic behavioral signal

## Abstract

Censored data occur commonly in trial-structured behavioral experiments and many other forms of longitudinal data. They can lead to severe bias and reduction of statistical power in subsequent analyses. Principled approaches for dealing with censored data, such as data imputation and methods based on the complete data’s likelihood, work well for estimating fixed features of statistical models but have not been extended to dynamic measures, such as serial estimates of an underlying latent variable over time. Here we propose an approach to the censored-data problem for dynamic behavioral signals. We developed a state-space modeling framework with a censored observation process at the trial timescale. We then developed a filter algorithm to compute the posterior distribution of the state process using the available data. We showed that special cases of this framework can incorporate the three most common approaches to censored observations: ignoring trials with censored data, imputing the censored data values, or using the full information available in the data likelihood. Finally, we derived a computationally efficient approximate Gaussian filter that is similar in structure to a Kalman filter, but that efficiently accounts for censored data. We compared the performances of these methods in a simulation study and provide recommendations of approaches to use, based on the expected amount of censored data in an experiment. These new techniques can broadly be applied in many research domains in which censored data interfere with estimation, including survival analysis and other clinical trial applications.

## INTRODUCTION

Observations are referred to as *censored* whenever the variable of interest is not completely observed, but some information about its value is nevertheless available (Berger, 2005). For example, a data point whose value is not known precisely but is known to be above some threshold is considered censored. Analysis methods for censored data have been of interest across a wide range of research areas, including medicine, biology, public health, epi demiology, engineering, economics, politics, demography, and neuroscience (Collins, 2006; Jones, Nagin, & Roeder, 2001; Klein & Moeschberger, 2005; Mihaylova, Briggs, O’Hagan, & Thompson, 2011; Prinja, Gupta, & Verma, 2010; Singh & Mukhopadhyay, 2011). Censored-data analysis methods are of particular interest in psychology studies, in which stimulus-evoked behavioral signals are analyzed to assess participants’ cognitive processes, decision making, and mental states (Budaev, 1997; Collins, 2006; Edelaar et al., 2012; Schafer & Graham, 2002).

Censored data may occur for a number of reasons. In many behavioral studies, the experimental design requires that the response window for each trial be limited in time, in which case the behavioral response is censored whenever it exceeds the time limit. For example, in the continuous-performance test (CPT), which is widely used to assess sustained and selective attention, the intertrial interval is limited in order to demand continuous preparation for rapid decision making. The behavioral response is censored whenever it is not received during the response window—referred to as an *omission error* (Riccio, Reynolds, Lowe, & Moore, 2002; Shalev, Ben-Simon, Mevorach, Cohen, & Tsal, 2011; van den Bosch, Rombouts, & van Asma, 1996). Another cause of censored data is insufficient dynamic range or resolution of the measuring apparatus. This can be related to sensor technology, such as clipping that occurs when an electroencephalography (EEG) sensor reads a signal outside its maximum measurement range, or can be due to the sampling resolution of a sensor or experimental procedure. For example, in clinical trials, patients are often seen in prescheduled visits, but the event of interest might occur anytime between the visits. If the event has not occurred at one visit (at time *L*), but has by the following visit (at time *R*), the time of the event is only known to be within the interval [*L*, *R*], and its true value is censored (Lindsey & Ryan, 1998). Finally, data might also be censored because of subject loss or dropout. For example, if cancer remission time is being studied and the patient is still in remission at the end of the study, then that patient’s remission time would be censored. If a patient for some reason drops out of a study before the end of the study period, then that patient’s follow-up time would also be considered to be censored (Bewick, Cheek, & Ball, 2004). The list of censored-data examples is abundant, and many more reasons can be listed as causes of censored data. Here, to develop our intuition, we will discuss censored data in terms of trial time limits. The developed methodologies, however, are broadly applicable to censored data generated by any of the aforementioned or any related reasons.

There are multiple reasons why an experiment might call for a time limit on the task trials. In many experiments, the amount of data to be collected is determined by statistical power considerations, but the amount of time available for the entire experiment is determined by practical considerations. In such experiments, the maximal individual trial duration may need to be limited in order to acquire a sufficient amount of data (Burock, Buckner, Woldorff, Rosen, & Dale, 1998). For example, Gale, Martinez-Rubio, Sheth, & Eskandar (2011) discussed limits to the intertrial interval for behavioral tasks in a surgical operating room, to capture a meaningful number of behavioral responses within the period of their experiment. Another experimental factor that could limit the response window is the fact that the behavioral response at long reaction times might reflect different underlying cognitive processes from the ones the experimenter intends to probe. Limited trial durations are embedded in the behavioral tasks to elicit the cognitive processes for which the response is a proxy (Lodge & Taber, 2005). For instance, in a choice-reaction time task (e.g., pressing a red or a green button), a participant might “lean” toward pressing the green button, and then start questioning his or her decision before pressing the green button. This phenomenon prompts a completely different cognitive process than when the participant has a limited time to respond and must press the green button after “leaning toward the green button” (Logan & Cowan, 1984). Similarly, in priming of cognitive tasks, participants are urged to respond as quickly as possible. In these tasks, a reaction time larger than 500 ms is rarely observed (Schmidt, Haberkamp, & Schmidt, 2011). For the same reason, in functional magnetic resonance imaging (fMRI) behavioral studies, where the interest is to link behavior to blood oxygenation level dependent (BOLD) responses, a standardized time window is assigned to each trial of a task in order to sample the same “assay” through consecutive trials (Buckner et al., 1998). On a related note, it is well known that time pressure can considerably affect decision making and behavior; a limited response window is embedded in many cognitive behavioral tasks in order to infer a meaningful insight about a participant’s capacity to perform the task. For example, in speed–accuracy tasks, the deadline method imposes an upper limit on reaction times (Wickelgren, 1977). There are also behavioral tasks in which the censored data themselves mark the behavior of interest. For example, in rapid visual information processing (RVP) and stop-signal tasks, nonrandom right-censored reaction times are valid measurements of the behavior being studied and are utilized to assess a participant’s attention (Coull, Frith, Frackowiak, & Grasby, 1996; Eagle et al., 2008).

Analysis of behavioral signals with censored values can present a statistical challenge, particularly when the censored trials do not occur completely at random, but instead are influenced by factors about which we would like to make inferences. One common approach to this problem is to remove trials on which the time limit was exceeded and then to perform statistical analyses on the remaining dataset. However, this can lead to increased estimation bias and variance for factors that influence the probability of exceeding the trial time limit (Graham, 2009). For instance, increasing the cognitive difficulty of certain trials may cause subjects to preferentially exceed the time limit on the most taxing trials, which may be the ones that also maximally stress the neural or psychological process of interest.

Here we will focus on a specific category of censored data, for which certain features are known—such as the fact that the time limit for a trial was reached—but other features are missing—such as what the response would have been had the trial been allowed to continue. We deal with censored data that fall in the category of “missing not at random” (NMAR; Little et al., 2012; Little & Rubin, 2014), in which the probability of a trial being censored depends on the values of the features to be measured. This is a common problem in many research fields, including engineering, economics, and medical research. Many of these research fields utilize specific techniques to deal with censored data (Allik, Miller, Piovoso, & Zurakowski, 2014; Ibarz-Gabardós & Zufiria, 2005; Sinopoli et al., 2004; Zheng, Niu, & Varshney, 2014). Nearly all, however, are based on a few distinct approaches, including data deletion, data imputation, and the complete likelihood, which fully accounts for the likelihood structures of both the observed and censored data. Many analyses use data deletion methods for simplicity, and in recent years such methods have been refined. The full maximum-likelihood (ML) and multiple-imputation (MI) methods have become state-of-the-art techniques for dealing with censored data (Baraldi & Enders, 2010; Enders, 2006; Schafer & Graham, 2002).

Even these advanced approaches have limitations. First, many commonly used techniques assume that the data come from continuous distributions or make the even stronger assumption that the data are normally distributed. In many behavioral experiments, the observed behavioral signals include both nonnormal continuous data—for instance, reaction times—and discrete variables—for instance, binary choice data (Coleman, Yanike, Suzuki, & Brown, 2011; Lacouture & Cousineau, 2008). Second, these techniques typically assume that data trials are independent and have properties that are stationary through time. In reality, subjects may experience practice effects, fatigue, and learning, all of which create nonstationarity in the reaction times. A common type of experiment applies a manipulation to subjects as they perform a task—for example, noninvasive brain stimulation to interfere with the cogni tive process of interest. These designs require an ability to infer the underlying dynamic in the presence of missing data, and these approaches have not yet been fully examined (Peugh & Enders, 2004). At the same time, behavioral studies designed to assess dynamic features of cognition, perception, and learning have been receiving increased interest. These experiments tend to generate multidimensional behavioral signals in which time-varying characteristics (including trial history effects) are of interest (Bush & Shin, 2006; Etkin, Egner, Peraza, Kandel, & Hirsch, 2006).

State-space methods are one way of expressing observed trial-level behaviors or physiologic phenomena as the output of an otherwise unobservable dynamical process. This approach has been used successfully to model dynamic features of behavioral signals (Byron et al., 2007; Prerau et al., 2009; Prerau et al., 2008; Smith & Brown, 2003; Srinivasan, Eden, Willsky, & Brown, 2006; Widge et al., 2017; Yousefi et al., 2015). Furthermore, state-space methods also play important roles in structural equation modeling and in estimating the parameters of dynamical-factor models. These models have a broad application in the analysis of multivariate psychological and biomedical time series data (Chow, Ho, Hamaker, & Dolan, 2010; Hamaker, Dolan, & Molenaar, 2005; Zhang, Hamaker, & Nesselroade, 2008). Classical state-space algorithms, such as the Kalman filter (Kalman, 1960; Roweis & Ghahramani, 1999), provide an optimal solution for tracking a linear-state process with normal stochastic com ponents using a linear observations process with normal noise terms. This methodology and its extensions have been used to model learning and attention (Dayan, Kakade, & Montague, 2000; Miltner, Braun, Arnold, Witte, & Taub, 1999). Recent advancements in state-space methods have also extended their applications to multivariate behavioral signals that are best fit using nonnormal probability distributions (Chen, Vijayan, Barbieri, Wilson, & Brown, 2009; Coleman et al., 2011; Fahrmeir, 1992; Molenaar & Newell, 2003; Yousefi et al., 2015).

Despite the existence of Bayesian filters and recursive estimators such as the Kalman filter for fully observed time series data, few methods are able to properly address the filter problem in time series data with missing or censored observations. Earlier proposed formulations used a Kalman filter with no measurement update on the missing data points (Kar, Sinopoli, & Moura, 2012; Sinopoli et al., 2004); this solution is only valid if the censored or missing data are uncorrelated with the state value. The method proposed in Ibarz-Gabardos and Zufiria (2005) derives a Kalman-like filter; the solution partitions the data into categories of censored and uncensored measurements and tries to combine these two data sources to estimate the state value. The formulation is not recursive and requires knowledge of the entire history of the censored data. The Tobit Kalman filter proposed by Allik et al. (2014) tries to estimate the expected mean and variance of the censored data points, then to use these measures in a Kalman-filter-type solution. The formulations used by both Allik et al. and Ibarz-Gabardos and Zufiria are only applicable to the class of linear-observation processes with normal noise terms, in which the goal is to derive a Kalman-like filter solution. Zheng et al. (2014) proposed a Bayesian framework for the filter solution with censored data in the detection system; their solution mixes a particle filter and a Kalman filter solution, and the censoring definition is different from the one presented here. Allik, Miller, Piovoso, and Zurakowski (2015) used a Monte Carlo method to compare the Tobit Kalman filter’s performance with the unscented and extended Kalman filter (UKF and EKF) methods, and demonstrated why the UKF and EKF methods provide unreliable estimates on censored data. Here, our focus was to demonstrate a unified framework that would address the censored-data problem in time series data for a larger class of observation processes, including mixtures of continuous and discrete signals. We used a Bayesian filter to address this problem, and we demonstrate the application of a Gaussian approximation method to derive an Kalman-like filter solution. We also demonstrate how using the Gaussian approximation method might provide a closed-form solution without requiring the computationally challenging calculations of the mean and covariance that are necessary in other methods.

In this article we develop a general state-space framework for estimating a dynamic state process in the presence of censored data. The state process evolves from trial to trial according to a defined state model, which may be linear with normal errors or nonlinear with more complicated error distributions. The evolution of the state process over the course of the experiment relates to changing influences on behavior that could be related to learning, attention, flexibility, or other cognitive states. The state-space model that we developed here can also be described as a hidden Markov model (Rabiner & Juang, 1986). Behavioral signals, such as those related to the time until either the completion of a trial or selection of a discrete response to a trial stimulus, are modeled using an observation process that characterizes the influence of the state on the association between stimulus and response. We used an exact filter algorithm to estimate the posterior distribution of the cognitive state process at each trial given the observed behavioral signals (Särkkä, 2013). We show that the three common approaches for dealing with censored data—data deletion, imputation, and computation of the complete data likelihood—can all be used to estimate dynamic signals under this single state-space framework. In addition to an exact formulation of the filter algorithm, we will also derive an approximate Gaussian filter (Eden, Frank, Barbieri, Solo, & Brown, 2004; Ito & Xiong, 2000; Truccolo, Eden, Fellows, Donoghue, & Brown, 2005), which can allow for more efficient estimation of the state process for high-dimensional state or observation processes.

In the Methods section, we first describe the general formulation of the filter algorithm for censored behavioral signals. We then derive a specific formulation for two example experimental scenarios. In the first scenario, the data come from a single continuous variable representing either the reaction time for a trial or the fact that a maximal reaction time was reached. In the second scenario, the data consist of both the reaction time and a binary variable representing whether a behavioral response was correct. The data in this scenario represent a mixture of continuous and discrete variables. In the Simulation Study section, we assess the properties of these filters and the relative merits of the data deletion, imputation, and full-likelihood approaches, using a simulation study. We use the root-mean squared error (RMSE) and the coverage of the 95% highest posterior density region (HPD) to quantify the accuracy of the estimates of the mean as well as the uncertainty of the state (Rosenkrantz, 1989). In the Discussion section, we conclude by discussing further work that will be needed in this field and possible future extensions of our own work.

## METHODS

### State-Space Model of Behavior

We utilized a state-space modeling approach to describe the dynamic features of behavioral data in trial-structured experiments. The model consists of a state equation and an observation equation. The state equation defines the temporal evolution of an unobserved process from one trial to the next, and the observation equation defines the influence of the unobserved process on the observed behavioral signals.

We assumed that the state variable is dynamic and changes from trial to trial through the course of the experiment; we call this variable the *cognitive state*. This state could relate to cognitive features such as attention or learning progression. We assume that the cognitive state is inaccessible to direct measurement and that its time evolution must be inferred from the observed behavioral signal(s). Let *x*_*k*_ be the value of the cognitive state at trial *k*, and assume that its evolution from trial to trial is defined by the conditional probability density function $f(\left.x_{k}\right|x_{k-1})$. Here we assume that the state model has the Markov property; thus, the probability distribution of the cognitive state at time *k* depends only on the cognitive state at time *k* − 1. Although this assumption simplifies the notation in the derivation, it is possible to extend these methods to non-Markovian processes (Cox, 1955).

The observation process defines the influence of the state process on the observed behavioral signals. For example, a higher inattention state might lead to longer reaction times and more incorrect responses. We consider two classes of observations. The first, *y*_*k*_, is a continuously valued signal with an upper bound *T*_*k*_, where the observation *y*_*k*_ may differ from trial to trial. If the value of this process exceeds the upper bound, the trial is censored, and features of the data are missing. Therefore, *y*_*k*_ takes on values in the set

$$\;y_{k}\in \left[0,T_{k}\right]\cup \left\{‘\text{Censored}’\right\}.$$ (1)

The second class of observations is defined by the random variable or vector *z*_*k*_. These are signals have values that are observed whenever $y_{k}\in \left[0,T_{k}\right]$, but missing whenever *y*_*k*_ = {“Censored”}. For example, *y*_*k*_might represent a reaction time, and *z*_*k*_ might be an indicator of a correct response in a binary choice trial. Trials with excessive reaction times are cut off, and neither the reaction time nor the response is observed. In experiments in which reaction time is the sole observed signal, *z*_*k*_ may be omitted. This class of censored data falls in the NMAR category; from this point onward, missing data are assumed to come from the NMAR class, except as otherwise mentioned (Little & Rubin, 2014).

We define $f\left(\left.y_{k}\right|x_{k}\right)$ to be the distribution of the observation process that can lead to censoring as a function of the state process. In cases in which additional signals may be missing during the censored trials, we define an additional observation model $f\left(\left.z_{k}\right|x_{k},y_{k}\right)$, which characterizes the distribution of *z*_*k*_ as a function of both the state process and the censored observation process *y*_*k*_. For each trial, we can compute the likelihood of any set of observations as a function of the state process:

$$\;L\left(x_{k};y_{k},z_{k}\right)=\left\{\begin{array}{ll} f\;\left(\left.z_{k}\right|x_{k},y_{k}\right)f\;\left(\left.y_{k}\right|x_{k}\right) & \;y_{k}\in \left[0T_{k}\right]\\ \text{Pr}\;\left(\left.y_{k}>T_{k}\right|x_{k}\right) & y_{k}=\left\{‘\text{Censored}’\right\}. \end{array}\right.$$ (2)

The objective is to estimate the distribution of the cognitive process *x*_*k*_ for each trial *k*, on the basis of the observed signals up to trial *k*.

### Exact Filter Algorithm

The exact filter computes the posterior distribution of the cognitive state *x*_*k*_ given the history of observations up to and including the current trial, *k*. The filter equations include two steps: (1) a prediction step and (2) an update step (Daum, 2005; Särkkä, 2013).

For the prediction step, the one-step prediction distribution of *x*_*k*_ given the observations up to but not including the current trial, is computed from the posterior filter distribution of the previous time step using the Chapman–Kolmogorov equation,

$$\;p\;\left(\left.x_{k}\right|y_{1:k-1},z_{1:k-1}\right)=\int f\;\left(\left.x_{k}\right|x_{k-1}\right)p\;\left(\left.x_{k-1}\right|y_{1:k-1},z_{1:k-1}\right)dx_{k-1},$$ (3)

where *y*_1:*k*−1_ and *z*_1:*k*−1_ are the observed behavioral signals up to trial *k* − 1. The expression $p\;\left(\;\left.x_{k}\right|y_{1:k-1},z_{1:k-1}\right)$ is the one-step prediction probability distribution function of *x*_*k*_ given the observations up to trial *k* − 1, and $p\;\left(\;\left.x_{k-1}\right|y_{1:k-1},z_{1:k-1}\right)$ is the posterior distribution of the state *x*_*k*−1_ given the observations up to time *k* − 1.

For the update step, the posterior distribution of the state *x*_*k*_ given the history plus the current observations (*y*_*k*_, *z*_*k*_) is computed using Bayes’s rule. The update rule for the observed data is defined by

$$\;p\;\left(\;\left.x_{k}\right|y_{1:k},z_{1:k}\right)\propto L\;\left(x_{k};z_{k},y_{k}\right)p\;\left(\;\left.x_{k}\right|y_{1:k-1},z_{1:k-1}\right).$$ (4)

$L\;\left(x_{k};z_{k},y_{k}\right)$ is defined by Equation 2, and $p\;\left(\left.\;x_{k}\right|y_{1:k},z_{1:k}\right)$ is the posterior distribution of *x*_*k*_ given the observations through time *k*.

Table 1 summarizes the recursive equations for one-step prediction and filtering at each time *k*. In this table, *p*(*x*_0_) expresses our belief about the state before the first trial; for example, *p*(*x*_0_) could be a delta function at a specific value, a uniform distribution over a certain range, or a normal distribution with known mean and covariance terms.

**Table 1.** Exact filter procedure

**Initialize:** Define the prior distribution of *p*(*x*_0_)

For each time *k*:

**Run prediction step:** Update the predictive distribution of *x*_*k*_ given the state model and previous
time posterior distribution.
$p\left(\left.x_{k}\right|y_{1:k-1},z_{1:k-1}\right)=\int f\left(\left.x_{k}\right|x_{k-1}\right)p\left(\left.x_{k-1}\right|y_{1:k-1},\;z_{1:k-1}\right)dx_{k-1}\;(5)$

**Run update step:** Update the posterior distribution of *x*_*k*_ given the observation *y*_*k*_ and the
predictive distribution.
$L\left(x_{k};\;y_{k},\;z_{k}\right)=\left\{\begin{array}{ll} f\left(\left.z_{k}\right|x_{k},\;\;y_{k}\right)f\left(\left.y_{k}\right|x_{k}\right) & y_{k}\in \left[0T_{k}\right]\\ \;\text{Pr}\left(\left.y_{k}>T_{k}\right|x_{k}\right) & y_{k}=\left\{‘\text{Censored}’\right\} \end{array}\right.\;(6)$
$p\left(\left.x_{k}\right|y_{1:k},z_{1:k}\right)\propto L\left(x_{k};z_{k},y_{k}\right)p\left(\left.x_{k}\right|y_{1:k‐1},z_{1:k‐1}\right)\;(7)$

The exact filter provides the full posterior distribution of the state given the sequence of observations up to the current trial. Using this distribution, we can compute any quantity of interest related to the state evolution. The numerical computations to calculate both the prediction and update steps of the exact filter can be performed using either simple Riemann summation (Jessen, 1934) or approximate methods such as Monte Carlo sampling (Hastings, 1970). The Riemann summation method is appropriate for problems with a scalar or low-dimensional *x*_*k*_, where a single small partition over the possible range of *x*_*k*_ often gives a fine approximation of the likelihood and posterior distribution. For a high-dimensional filter problem, the size of the partition required to build an accurate approximation of the likelihood and posterior distribution grows exponentially with the *x*_*k*_ dimension; thus, approximate methods like Monte Carlo might be a more computationally efficient solution. In the next section, we illustrate the filter solution for an example state-space model with censored data.

### Example Problem Specification

Here we give example models for the state and observation equations and compute the corresponding likelihoods and distributions for the filter algorithm. Imagine a sustained-attention-to-response task, similar to the ones discussed by Lesh et al. (2013) and Riccio et al. (2002), in which the participant has a limited amount of time on each trial to respond. In our example, a simulated participant is required to respond in a limited time window (*T*_*k*_) after stimulus presentation; otherwise, the task moves to the next trial. For example, in an A–X CPT task, the subject is instructed to respond with a right mouse-press whenever the stimulus is an X that was preceded by an A. The left mouse button is pressed for all other stimuli, including an A, an X that was not preceded by an A, and any other letter. We assume that both the reaction time and the outcome of the decision are affected by the subject’s attention/inattention state. Let the state variable *x*_*k*_ represent an inattention level, and let *y*_*k*_ and *z*_*k*_ be the reaction time and the decision result for the *k*th trial of the task.

In this example, we model the subject’s inattention level as a first-order autoregressive—AR(1)—process,

$$\;\;\;\;\;\;x_{k}=a_{1}x_{k-1}+a_{0}+\varepsilon_{k}.$$ (8)

The parameters *a*_0_ and *a*_1_ define the dynamics of the inattention state through the course of the experiment, whereas the process noise term, *ε*_*k*_, represents unobserved perturbations that cause the subject’s inattention level to go up or down. If 0 < *a*_1_ < 1, then the steady-state expected value of the inattention state is $a_{0}/\;\left(1-a_{1}\right)$. We assume that *ε*_*k*_ has a zero-mean Gaussian distribution with variance $\sigma_{\varepsilon }^{2}$; therefore, $f(\left.x_{k}\right|x_{k-1})$ is a Gaussian distribution with mean *a*_1_*x*_*k*−1_ + *a*_0_ and variance $\sigma_{\varepsilon }^{2}$.

We model the relationship between reaction time *y*_*k*_ and inattention level *x*_*k*_ as

$$\;\;\;\;\;\;\;\log y_{k}=b_{1}x_{k}+b_{0}+v_{k},$$ (9)

where the logarithm of reaction time is defined by a linear function of the state *x*_*k*_ plus an additive Gaussian noise process *v*_*k*_. Parameters *b*_0_ and *b*_1_ define the relationship between the reaction time and the inattention state, and the additive noise *v*_*k*_ has a Gaussian distribution with zero mean and variance $\sigma_{v}^{2}$. For this model, exp(*b*_0_) is the expected reaction time when the state has a value of zero, and exp(*b*_1_) determines how the expected reaction time is scaled in response to a unit change in the inattention state. If the *b*_1_ term is positive, the reaction time will tend to increase as the state value increases. The reaction time will be censored if the subject’s response takes longer than *T*_*k*_. Here, we define the logarithm of the reaction time as a linear function of *x*_*k*_. Experimental results suggest that either the Gamma or the log-normal distribution provides a better fit than the normal distribution to the reaction times recorded in these behavioral signals (Yousefi et al., 2015).

We model the binary response process *z*_*k*_ as a Bernoulli process in which the probability of a correct response given the attention state, *p*_*k*_ is given by

$$\;\;\;\;\;\text{logit}(p_{k})=c_{2}x_{k}+c_{1}y_{k}+c_{0}.$$ (10)

Here, a positive *c*_1_ suggests that longer reaction times lead to a higher probability of a correct trial, and a negative *c*_2_ suggests that the probability of a correct trial decreases as inattention level increases. For this model, exp(*c*_0_)/[1 + exp(*c*_0_)] determines the probability of a correct trial when both the inattention state and reaction time values are zero. When the reaction time is longer than *T*_*k*_, the binary response is also censored. The model of the response process defined here assumes a particular dependence between the decision and both our inattention state and the other covariate term *y*_*k*_ (in this case, the observed reaction time). In general, the proper choice for models of the reaction time or decision depends on task factors and the variables of interest.

The full likelihood of the observations for the *k*th trial is therefore expressed by Equation 11,

$$L\left(x_{k};y_{k},z_{k}\right)\;=\;\left\{\begin{array}{ll} \frac{1}{\sqrt{2\pi \sigma_{\nu }^{2}}}e^{-\frac{{\left(\log y_{k}-b_{1}x_{k}-b_{0}\right)}^{2}}{\left(2\sigma_{v}^{2}\right)}}{\left(e^{c_{2}x_{k}+c_{1}y_{k}+c_{0}}\right)}^{z_{k}}\left(\frac{1}{1+e^{c_{2}x_{k}+c_{1}y_{k}+c_{0}}}\right)\;\;\;\;\;\;\;\;\;\;\;\;\; & y_{k}\in \left[0T_{k}\right]\\ \frac{1}{2}-\frac{1}{2}\phi \left(\frac{\log T_{k}-b_{1}x_{k}-b_{0}}{\sigma_{v}\sqrt{2}}\right) & y_{k}=\left\{‘\text{Censored}’\right\}, \end{array}\right.$$ (11)

where *ϕ* represents the error function for a standard normal distribution. The likelihood function suggests that the probability of observing a censored reaction time increases as the in attention state *x*_*k*_ increases when *b*_1_ is positive.

Equation 11 defines the complete likelihood function of the observed behavioral signal given the state variable. Using the exact filter technique—defined by Equations 5 and 6—we are able to compute the posterior distribution, $p\;\left(\;\left.x_{k}\right|y_{1:k},z_{1:k}\right)$.

Here we assume that the model parameters described in Equations 8, 9, and 10 are known and fixed. In the Discussion section, we will briefly discuss issues related to parameter estimation (Ghahramani & Hinton, 1996; Prerau et al., 2009; Titterington, 1984); we note, however, that the full solution to this problem is beyond the scope of this article.

### Gaussian Approximation

The exact filter is computationally tractable for low-dimensional state processes, but it can become computationally expensive when the state process becomes higher-dimensional. For the example problem described here, the state is one-dimensional, representing a single cognitive feature (attention). The state process can also represent a multivariate factor. For example, in learning tasks, subjects are often required to learn multiple arbitrary associations simultaneously (Asaad & Eskandar, 2011). Multidimensional learning state processes could then represent the probability of a correct response to each association at a given time point. If that is the case, we can derive a computationally efficient approximate filter by assuming that the posterior distribution is approximately Gaussian at each time step. In that case, we need only compute a mean and a covariance at each time step to describe the posterior distribution. This Gaussian approximation is appropriate for a large class of state and observation models in which the distributions are not heavy-tailed and the true posteriors are unimodal and symmetric at all time steps (Eden & Brown, 2008).

Let $x_{\;\left.k\right|k}=E(x_{k}|y_{1:k}z_{1:k})$ and $\sigma_{k|k}^{2}=Var(x_{k}|y_{1:k},z_{1:k})$ be the mean and variance of the posterior distribution, and let $x_{\;\left.k\right|k-1}=E(x_{k}|y_{1:k-1},z_{1:k-1})$ and $\sigma_{k|k-1}^{2}=Var(x_{k}|y_{1:k-1},z_{1:k-1})$ be the mean and variance of the one-step prediction distribution, respectively. If we assume that the posterior distribution of the previous trial is Gaussian, then simple integration of the Chapman–Kolmogorov Equation 7 shows that the one-step prediction distribution will also be Gaussian and that the means and variances are related as follows:

Mean and variance of one-step prediction for a first-order autoregressive process

$$\;\;\;\;\;x{\;}_{\left.k\right|k-1}=a_{0}+a_{1}x_{\left.k-1\right|k-1},$$ (12)

$$\;\;\;\;\;\sigma_{k|k-1}^{2}=a_{1}^{2}\sigma_{k-1|k-1}^{2}+\sigma_{\epsilon }^{2}.$$ (13)

We compute the Gaussian approximation of the posterior distribution at the current time step by expanding its logarithm using a second-order Taylor expansion about the one-step prediction mean from the previous trial. We then compute the mean and variance of the approximate Gaussian posterior distribution. This gives iterative expressions for the poste rior mean, $x{\;}_{\left.k\right|k}=E(x_{k}|y_{1:k},z_{1:k})$, and the posterior variance, $\sigma_{k|k}^{2}=Var(x_{k}|y_{1:k},z_{1:k})$. For the Example Problem Specification section, when the state model is given by Equation 8 and the only observation process is a reaction time given by Equation 9, the resulting iterative expressions for the approximate Gaussian filter are as follow:

Approximate Gaussian filter for continuous-valued observation with censored data

$$\;{ŷ}_{k}=b_{1}x_{\left.k\right|k-1}+b_{0},$$ (14)

$$\;\;e_{k}=\left(\log y_{k}I_{k}+\left(1-I_{k}\right)\log T_{k}\right)-{ŷ}_{k},$$ (15)

$$\;\varphi_{k}=1/\;\left(\sigma_{v}\sqrt{2\pi }\right)exp\;\left(-e_{k}^{2}/2\sigma_{v}^{2}\right),$$ (16)

$$\;L_{k}=1/2-1/2\;\phi \left(e_{k}/\;\left(\sigma_{v}\sqrt{2}\right)\right),$$ (17)

$$\;U_{k}=\varphi_{k}/L_{k},$$ (18)

$$\;x_{\left.k\right|k}=x_{\left.k\right|k-1}+b_{1}\sigma_{\left.k\right|k}^{2}\left[I_{k}e_{k}+\left(1-I_{k}\right)U_{k}\right],$$ (19)

$$1/\sigma_{\left.k\right|k}^{2}=1/\sigma_{\left.k\right|k-1}^{2}+b_{1}^{2}/\sigma_{\varepsilon }^{2}\left[I_{k}+\left(1-I_{k}\right)U_{k}^{2}\left(\sigma_{\varepsilon }^{2}-e_{k}/U_{k}\right)\right],$$ (20)

where the indicator variable *I*_*k*_ is 1 when the reaction time is observed and 0 when the reaction time is censored. In Equation 14, ${ŷ}_{k}$ is the predicted reaction time for the *k*th trial, and *e*_*k*_ is the measurement residual error. The variables *φ*_*k*_ and *L*_*k*_ are the probability density function and cumulative distribution function of ${ŷ}_{k}$ for a Gaussian distribution with mean $b_{1}x_{\left.k\right|k-1}+b_{0}$ and variance $\sigma_{v}^{2}$. The term $x_{\left.k\right|k}$ is the estimate of the posterior mean, and $\sigma_{\left.k\right|k}^{2}$ is the estimate of the posterior variance. The terms $x_{\left.k\right|k-1}$ and $\sigma_{\left.k\right|k-1}^{2}$ are the mean and variance of the one-step prediction distribution. Appendix B contains a step-by-step derivation of the approximate filter (Yousefi, Dougherty, Eskandar, Widge, & Eden, 2017).

If we add the Bernoulli observation process for the binary choice task also described in the Example Problem Specification section, we modify the approximate Gaussian filter above by removing Equations 19 and 20, and replacing them with the following equations:

Gaussian approximate filter for the reaction time and binary decision observation with censored data

$$\;{q_{\left.k\right|k-1}=e}^{c_{2}x_{\left.k\right|k-1}+c_{1}y_{k}+c_{0}}/\;\left(1+e^{c_{2}x\left.k\right|k-1+c_{1}y_{k}+c_{0}}\right)$$ (21)

$$\;d_{k}=z_{k}-q_{\left.k\right|k-1}$$ (22)

$$\;x_{\left.k\right|k}=x_{\left.k\right|k-1}+\sigma_{\left.k\right|k}^{2}\left[I_{k}\left(b_{1}e_{k}+{d_{k}c}_{2}\right)+\left(1-I_{k}\right){b_{1}U}_{k}\right]$$ (23)

$$\begin{array}{c} \;1/\sigma_{\left.k\right|k}^{2}=1/\sigma_{\left.k\right|k-1}^{2}+\left[I_{k}\left(b_{1}^{2}/\sigma_{\varepsilon }^{2}+c_{2}^{2}q_{\left.k\right|k-1}\left(1-q_{\left.k\right|k-1}\right)\right)\right.\\ \;+\left(1-I_{k}\right)b_{1}^{2}/\sigma_{\varepsilon }^{2}U_{k}^{2}1\;\left(\sigma_{\varepsilon }^{2}-e_{k}/U_{k}\right)] \end{array}$$ (24)

where $z_{k}\in \left\{0,1\right\}$ is the decision result of the observed data. Note that whenever a trial is censored, only the reaction time contributes to the updated state estimate. This is because the decision result for the censored data points does not give any information about the state.

## SIMULATION STUDY

We performed a simulation study to examine the performance results for four different estimation algorithms for the censored-data example problem described in the Example Problem Specifications section. In addition to the exact filter for censored data and the Gaussian approximate filter described above, we modified the exact filter to implement commonly used data deletion and data imputation approaches to missing-data problems; these techniques will be described in the following subsections. The comparison criteria for these approaches are the root-mean square error (RMSE) between the true simulated state process and its estimate, as well as the coverage of the 95% highest probability density (HPD) region of the computed posterior distribution (Hyndman, 1996; Rosenkrantz, 1989). We generated 500 realizations of the cognitive state and observation signals for each simulation scenario. We first define the simulation procedure of the cognitive state and the observation signals. We then discuss the various algorithms’ performance in detail.

### Simulation Process: Cognitive State and Observation Signal

Each of the 500 realizations of the state and observation processes included 100 trials of data. The cognitive state was modeled using Equation 8 with parameters *a*_1_ = 0.95 and *a*_0_ = 0.025. The process noise at each time point had an independent and identically distributed (i.i.d.) Gaussian distribution with zero mean and standard deviation 0.078. The steady-state mean and standard deviation of the cognitive state, in the absence of any observations, would be 0.5 and 0.25, respectively. This means that the cognitive state will be within the interval [0 1] with high probability. We interpret *x*_*k*_ as an inattention state (rather than an attention state), such that increasing values of *x*_*k*_ represent increasing inattention to the task and decreasing performance.

The reaction time was modeled by Equation 9 with parameters *b*_1_ = 1 and *b*_0_ = −0.6. The observation noise was an i.i.d. Gaussian process with zero mean and standard deviation 0.141. For these parameters, the expected reaction time is a monotonically increasing function of the cognitive state. The binary response was modeled using Equation 10 with parameters *c*_1_ = 10, *c*_2_ = −8.5, and *c*_0_ = −3.5. The fact that *c*_1_ is positive suggests that trials with longer reaction times are more likely to be correct. Similarly, the fact that *c*_2_ is negative suggests that when the subject’s inattention level increases, the probability of a correct response decreases.

We examined the performance of all the estimation algorithms as we varied the censoring threshold over the full range of observed reaction times—which is assumed to be from 0.2 to 2 s. Note that as the censoring threshold decreases, the expected number of missing data points grows.

### Alternative Estimation Approaches

Data deletion and data imputation methods are widely used techniques to deal with missing data. For the state-space filter problem, it is possible to express both of these approaches for dealing with missing data using Equations 5 and 6, by replacing the likelihood function $L\left(x_{k};z_{k},y_{k}\right)$. One can remove the influence of trials with missing data in the first case, or alter the function to reflect the likelihood of imputed data in the second case. The details for implementing these approaches within our filter algorithm are detailed in the following two sections.

**Data deletion** Typically, the data deletion approach involves removing any trials with censored data from the dataset before analysis. For dynamic, state-space models, when removing trials, we still need to account for possible changes in the state process that might occur during the removed trials. In the filter framework in Equations 5 and 6, this is handled by the one-step prediction in Equation 5. To remove the influence of the censored trials’ observations, we replace the likelihood in Equation 6 with a new likelihood that is identical for any trial that is not censored, and is set to 1 for any censored trials. Mathematically, this is stated in Equation 25.

$$L\left(x_{k};y_{k},z_{k}\right)\;=\;\left\{\begin{array}{ll} \frac{1}{\sqrt{2\pi \sigma_{\nu }^{2}}}e^{-\frac{{\left(\log y_{k}-b_{1}x_{k}-b_{0}\right)}^{2}}{\left(2\sigma_{v}^{2}\right)}}{\left(e^{c_{2}x_{k}+c_{1}y_{k}+c_{0}}\right)}^{z_{k}}\left(\frac{1}{1+e^{c_{2}x_{k}+c_{1}y_{k}+c_{0}}}\right) & y_{k}\in \left[0T_{k}\right]\\ 1 & \;\;\;\;\;\;\;\;\;\;y_{k}=\left\{‘\text{Censored}’\right\}\;. \end{array}\right.$$ (25)

**Data imputation** In contrast to the data deletion technique, which completely removes the influence of trials with missing data, the data imputation technique tries to predict and substitute for missing data. One possible method for data imputation is sampling the missing components of the data from the best estimate of the population distribution, conditioned on the observed components of the data (Raghunathan, Lepkowski, Van Hoewyk, & Solenberger, 2001; Schafer & Graham, 2002). For this method, the sampling procedure at each missing time point is defined by the following procedure:

1.  Draw a sample from the one-step prediction distribution ${\tilde{x}}_{k}∼\left.x_{k}\right|{\tilde{y}}_{1:k-1},{\tilde{z}}_{1:k-1}$ (Equation 6).
2.  Draw a sample from ${\tilde{y}}_{k}∼\left.y_{k}\right|{\tilde{x}}_{k}$ given the sample from the first step $\left({\tilde{x}}_{k}\right)$.
3.  If the sample from the second step $\left({\tilde{y}}_{k}\right)$ is smaller than *T*_*k*_, return to Step 1 to resample $\left({\tilde{x}}_{k},{\tilde{y}}_{k}\right)$.
4.  If the sample from the second step $\left({\tilde{y}}_{k}\right)$ is longer than *T*_*k*_, draw a sample from ${\tilde{z}}_{k}∼\left.z_{k}\right|{\tilde{x}}_{k},{\tilde{y}}_{k}$—Equation 10.
5.  The variables ${\tilde{y}}_{k}$ and ${\tilde{z}}_{k}$ are the imputation samples.

Note that, for any trials that are not censored, we set ${\tilde{y}}_{k}=y_{k}$ and ${\tilde{z}}_{k}=z_{k}$ to be the observed values. Thus, the filtering process will run at each step with the new data set $\left({\tilde{y}}_{k},\;{\tilde{z}}_{k}\right)$. It is common to run this imputation technique multiple times, which generates multiple realizations of the missing data; the filter solution can be computed separately for each imputation, or once using a product likelihood over multiple imputations. Here, we examined the performance using multiple imputations on each missing data point. Per each missing data point, we draw *m* samples—*m* is 10 in the simulation—and the filter solution is the average filter solution given each drawn sample (Little & Rubin, 2014). Using the drawn sample, the filter is computed as in Equations 5 and 6, with a likelihood given by

$$L\left(x_{k};{\tilde{y}}_{k},{\tilde{z}}_{k}\right)={\frac{1}{\sqrt{2\pi \sigma_{\nu }^{2}}}e}^{-\frac{{\left(\log{\tilde{y}}_{k}-b_{1}x_{k}-b_{0}\right)}^{2}}{\left(2\sigma_{v}^{2}\right)}}{\left(e^{c_{2}x_{k}+c_{1}{\tilde{y}}_{k}+c_{0}}\right)}^{{\tilde{z}}_{k}}\left(\frac{1}{1+e^{c_{2}x_{k}+c_{1}{\tilde{y}}_{k}+c_{0}}}\right)\;.$$ (26)

Note that this is identical to the likelihood in Equation 11, except that the data values *y*_*k*_,*z*_*k*_ have been replaced with the sequence of observed and imputed values ${\tilde{y}}_{k},{\tilde{z}}_{k}$. For missing-data trials, the imputed values are consistent with the observation that the reaction time is above the censoring threshold.

### Methods of Comparison

We characterized the performance of the different algorithms using two metrics. The first metric calculates the RMSE between the mean of the state estimate and its known simulated value. The second metric counts the number of trials for which the simulated state value falls in the 95% HPD region (Hyndman, 1996; Tanner, 1991) of the estimated posterior distribution from the filter procedure. If the filter estimate is accurate, we would expect 95% of trials to fall in this region. For a state with an approximately Gaussian posterior distribution, the HPD region is $\left[x_{\left.k\right|k}-1.96{\;\sigma }_{\left.k\right|k},x_{\left.k\right|k}+1.96{\;\sigma }_{\left.k\right|k}\right]$. For this simulation problem, the true posterior distribution is non-Gaussian; thus, the 95% HPD region was computed numerically. We measured the performance over 500 realizations of state models, each comprising 100 trials, for possible censoring threshold values. As the threshold values decreased, the fraction of trials that were censored increased. We visualized the performance results as a function of the expected fraction of data that were missing for each threshold value.

### Results

**Figure 1.**
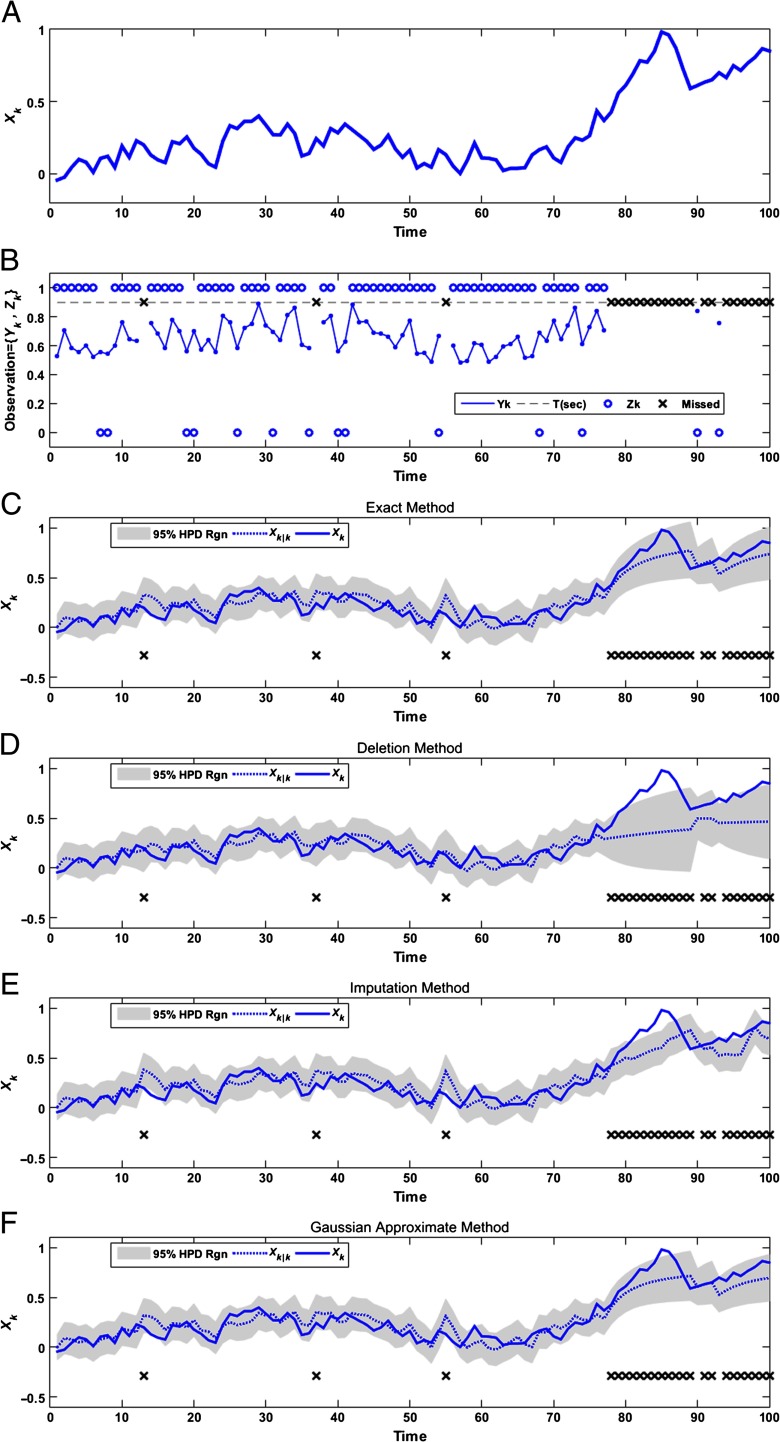
**Estimation results for a single realization.** (a) One realization of the inattention-state process over 100 trials, based on Equation 8. (b) Realization of reaction time and binary decision data, based on the state process in panel a and Equations 9 and 10. Both the reaction time and binary response data are censored whenever the reaction time is longer than 0.9 s. The circles show the binary decision, either 0 or 1, and the “x” marks show the missing points. (c) Estimation results for the exact filter, based on the full likelihood in Equation 11. (d) Estimation results for data deletion, based on the likelihood in Equation 25M. (e) Estimation results for data imputation, based on the likelihood in Equation 26. (f) Estimation results for the approximate Gaussian filter, detailed in Equations 21–24.

Figure 1 shows an example of each filter methodology applied to a single realization of the simulated cognitive state and observation process, each comprising 100 simulated trials. Figure 1a shows the true cognitive state to be estimated by the filter algorithms. The cognitive state fluctuates between –0.045 and 0.982; it grows to 0.982 at the end of Trial 85, and its minimum occurs on the first trial.

Figure 1b shows the simulated reaction times and binary responses and indicates censored trials with an “x” label. The circle labels represent the binary decision—either 0 or 1, where circle labels with value 1 correspond to a correct response. Since *b*_1_ is positive, the reaction time is positively correlated with the unobserved state process. For this example, the threshold for censoring was any reaction time exceeding 900 ms. Some censored trials occur in isolation, whereas others occur in sequence; for example, at Trials 13, 37, and 55 the reaction time jumps above the censoring threshold for just one trial, whereas the entire sequences of trials between 78 and 89, and similarly between 94 and 100, are censored. The binary decision tends to be more correct at the beginning of the session and around Trials 42 through 67, where the state process is smaller. Note that the number of censored trials increases as the inattention state increases.

Figure 1c to1f show the state estimates and HPD bounds using each of the different approaches described above to deal with the missing data. For each panel, the true state process is shown with a solid line, the mean of the posterior estimate for each filter is shown with a dotted line, and the 95% HPD region is shown in gray. Figure 1c shows the estimation result for the exact filter using the full likelihoods of both the observed and censored trials; the estimate of the state process tends to follow the true process, even during sequences of trials that are censored. During these missing-data sequences, the variance of the posterior tends to increase, shown by the widening of the HPD region, to reflect the increasing uncertainty about the state due to the missing data. For this realization, the HPD region contained the true value of the state for 96 of the 100 trials—close to the expected 95%.

Figure 1d shows the estimation result for the data deletion method. Under the state-space framework of Equation 8, the posterior distribution of the state is updated during censored trials by one-step prediction based on the state dynamics in Equation 9. There is, however, no additional information based on the data likelihood. For trials with missing data, such as the sequence from Trials 78 to 89, the mean moves slowly, on the basis of the state dynamics, but does not use the knowledge that censored trials are more likely to occur when the state is high. Therefore, the data deletion estimator tends to underestimate the state during the censored trials. For those same trials, the confidence interval defined by the HPD region increases in width, accurately reflecting the increasing uncertainty in the estimator due to the missing data. In this example, the HPD region still covers the true state value during the first few missing-data trials, such as with Trials 78 and 79, but it fails to cover the true state as the number of missing-data trials in sequence increases, such as during Trials 80 through 87. For this realization, the HPD region contained the true value of the state for only 87 of the 100 trials.

**Figure 2.**
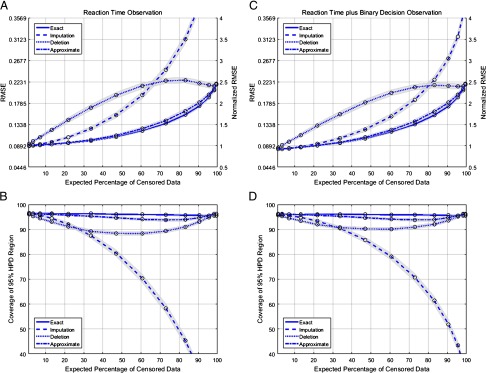
**Estimator accuracy summary results.**(a) Root-mean square error (RMSE) values as a function of the expected fraction of censored trials, based only on reaction time data. (b) Fractions of trials in which the true state value is contained in the estimated 95% HPD region, as a function of the expected fraction of censored trials based only on reaction time data. (c) RMSE values as a function of the expected fraction of censored trials, based on both reaction time and binary decision data. (d) Fractions of trials in which the true state value is contained in the estimated 95% HPD region, as a function of the expected fraction of censored trials, based on both reaction time and binary decision data. The simulation was run for a set of threshold values marked by “o,” and the performance graphs are derived by interpolation of the corresponding marked points.

Figure 1e shows the estimation results using the data imputation method. Under the state-space framework in Equation 8, each missing data point is replaced by a randomly generated sample of the reaction time and a binary decision based on the best current estimate of the state. The sampling procedure was described in detail in the Data imputation section. The estimates using the imputed data show a lower bias than with the deletion method during sequences of censored trials that were apparent in the data deletion estimate (e.g., from Trials 78 to 89). When the amount of missing data is small, accurate state estimations from previous trials lead to good imputed data values, which helps preserve the accuracy of the state estimate during censored trials. For example, the HPD region covers Trials 78 to 81, whereas the data deletion HPD region includes only Trials 78 and 79. We will show later—in Figure 2, using additional simulations—that this can break down when the fraction of censored trials becomes large. Another major difference between the data imputation and data deletion methods is that the width of the HPD region does not show the same marked increase during sequences of missing data. This widening should be present to accurately reflect the fact that the estimator should not be as confident in these regions. This suggests that the data imputation method could lead to misleading overconfidence about the accuracy of the estimates. For this realization, the estimated HPD region contained the true state value for only 85 of 100 trials; we will see later in this section that the data imputation method often leads to an incorrect inference of confidence, even when the amount of missing data is small. For example, for the missing Trial 55, the filter overestimates the state variable in both the exact and imputation methods. However, the smaller HPD region for the imputation method fails to cover the true state, whereas the exact HPD region does cover it.

Figure 1f shows the estimation results using the approximate Gaussian filter for the exact posterior that is defined in Equations 21–24. In this case, the results look nearly identical to the numerical computations of the exact filter shown in Figure 1c. This is not surprising, since the posterior computed for the exact filter is unimodal and approximately symmetric, and could be approximated well by a Gaussian distribution. However, during sequences of censored trials, the exact posterior becomes increasingly positively skewed. When the fraction of missing data becomes large, the quality of the Gaussian approximation may decrease. The exact posterior distributions of the missing trials from 85 to 89 and similarly from 96 to 100 are positively skewed, whereas the HPD region based on the Gaussian approximate is always symmetric. This leads to erroneous bounds for Trial 85, where the true value is not covered by its estimated HPD region. When the state model is high-dimensional, the approximate Gaussian filter may be a preferred option, since its estimate is comparable to that of the full exact filter at a reduced computational cost. For this example, the HPD region derived by the Gaussian approximate method contains the true value of the state for 95 of the 100 trials—which is equal to the expected 95%.

Figure 2 shows a summary of RMSE and the coverage of the HPD region over 500 realizations for each of the filter approaches as a function of the expected percentage of censored trials. Figures 2a and 2b show the results under a simulation scenario in which the observation process includes only the reaction time, generated according to Equation 9. Figures 2c and 2d show the results for the simulation scenario in which the observation process includes both the reaction time and binary decision data, generated according to Equations 9 and 10. The expected percentage of trials with missing data grows as we decrease the censoring threshold.

In both simulation scenarios, the exact filter produces the lowest RMSE and maintains the state estimate within the 95% HPD region, independent of the expected fraction of censored trials. For the log-normal observation, the minimum error value when no data are missing should be the solution of the Riccati equation, which is a function of both the process noise and observation noise (Assimakis & Adam, 2013). For the example problem, the expected RMSE for fully observed data is 0.089. The maximum error value for fully censored data is the steady-state standard deviation of the state process; the expected RMSE for fully censored data will be 0.25. For the mixed behavioral signal, the solution of the Riccati equation gives the upper bound of the expected minimum error. Because of the information carried by the binary observation, the equivalent observation process noise is lower for the mixed behavioral signal. Despite the decrease in the expected minimum error, the maximum error value for fully censored data using the mixed behavior signal will be equal to that of the log-normal observation process alone. When there is no censoring, the RMSEs for both sets of observation processes approach their respective Riccati equation solutions: 0.0892 for reaction time alone, and 0.0815 for the mixed observation process. The RMSE for fully censored data reaches the steady-state standard deviation in the absence of observations, 0.25. Figures 2a and 2c also show raw and normalized values for the RMSE, where the normalization sets to 1 the expected fully observed error based on the solution to the Riccati equation—in order to provide a better picture of the different methods’ RMSEs with respect to the expected minimum error.

The approximate Gaussian filter is nearly as accurate as the full-likelihood filter in terms of RMSE and has only slightly reduced coverage of the HPD region across the entire range of values for the expected fraction of censored trials. We expect the approximate Gaussian filter to be less accurate in situations in which the true posterior is skewed. For example, when there is a large run of censored trials and the state wanders far above the threshold, the exact posterior will become skewed and its HPD region will tend to cover the true state, whereas the approximate Gaussian filter cannot model this skew. We do indeed see a slight performance degradation in the approximate Gaussian filter when a very high fraction of trials are censored, but this difference is minor relative to those from the other approaches examined. The data imputation approach performs nearly as well as the exact and approximate Gaussian filters, in terms of both RMSE and HPD coverage, in cases in which the expected fraction of missing data is small—for example, below 10% of trials. For many experimental paradigms, the expected fraction of missing data is well below this range, suggesting that the data imputation approach is likely to work well in practice. In cases in which the expected fraction of censored trials becomes large, we see the data imputation filter performance degrade in two ways. First, the difference between its RMSE and that of the exact filter increases consistently with increasing censoring. This can be explained by mounting errors in the state estimation due to the incorporation of increasing numbers of inaccurate imputed values. When long sequences of missing data occur, this filter can have substantial errors. Second, we see a dramatic decrease in the HPD coverage of the true state value as the fraction of missing trials becomes large. This is likely due to the fact that the estimated uncertainty is artificially small. The imputation filter does not “know” which data were actually observed versus imputed, and it weights all observations equally. This assigns an undue weight to the imputed and uncertain observations and creates a false confidence. When the RMSE of the estimate starts to increase, the resulting artificially small HPD region becomes much less likely to cover the true state value. In contrast, the performance of the data deletion approach begins to degrade almost immediately as the number of trials with missing data increases. Initially, the RMSE increases linearly with the expected fraction of censored trials, eventually reaching a point nearly three times greater than that of the exact filter. This is due to the fact that censored trials do contain useful information about the state process that is thrown away by the data deletion approach. In this example, that information is the fact that larger values of the inattention state are more likely to lead to a censored trial. As the censoring threshold decreases and the expected number of censored trials increases, the amount of information available in censored trials also decreases. This explains the eventual convergence of the RMSEs for the data deletion and exact-filter approaches when the fraction of trials with missing data nears 100%. Similarly, the coverage of the 95% HPD region for the data deletion filter initially decreases linearly, due to the bias in the estimate. Only when the fraction of censored trials approaches 100% does the coverage return to the correct value near 0.95. Of course, for most real experiments, the amount of missing data will be far below these levels.

Comparing the simulation results between the scenario in which the observations are limited to the reaction times alone and the scenario in which the observations include both the reaction time and binary decisions, we find that when no data are missing, the RMSE for the latter scenario is reduced. The binary data provides additional information about the state process. For small amounts of missing data, the RMSE for the imputation method follows the exact filter better than the scenario in which only reaction times are observed. This can be attributed to the fact that both the reaction time and binary decision data are being imputed simultaneously, and the binary decision contributes to better estimates of the state value. The data imputation method outperforms the data deletion method for a significant range of expected percentages of censored data. Examining the coverage of the HPD regions, we find that the data imputation method is again more likely to overestimate its certainty, and therefore less likely to cover the true values when both the reaction time and the binary decision must be imputed. The coverage probability drops substantially in comparison to the data deletion method as the amount of missing data passes about 30%.

## DISCUSSION

We have developed a filter paradigm to estimate the evolution of a dynamic state process that influences behavioral responses in sequential trial data, in the presence of missing data due to censored trials. We showed that this single paradigm encompasses the three distinct approaches often used to account for missing data—data deletion, data imputation, and computation of the full likelihood—by modifying the likelihood term in Equations 5 and 6. Additionally, we developed an approximate Gaussian filter for censored data, which requires less computational effort to solve for high-dimensional state processes.

We performed a simulation study to compare the performance of these four different approaches, in terms of both estimator quality and the accuracy of their uncertainty estimates, over a range of missing-data scenarios. Overall, we found that for even small amounts of missing data, the data deletion approach led to substantial bias and could lead to improper inferences related to inaccurate measures of uncertainty. For small to moderate amounts of missing data, the data imputation approach performed nearly as well as the exact filter, but as the number of missing trials became large, the data imputation approach began to perform poorly. Its confidence regions eventually had worse coverage of the true state than even the data deletion approach. Although the exact filter had the best performance overall, in terms of both minimizing bias and correct coverage of the confidence regions, the approximate Gaussian filter performed nearly as well, even with a large number of censored trials.

The performance of each approach can be understood on the basis of the characteristics of the posterior distributions they compute, in particular during long sequences of missing data. For any censored trial, the mean of the posterior from the exact filter tends to move in a direction that increases the probability of censoring, the variance tends to increase due to the missing data, and the skewness tends to increase in the direction that makes censoring more likely. In our simulation example, since the *b*_1_ term in Equation 9 was positive, on each censored trial that extended beyond the maximum reaction time, the estimate of the inattention state from the exact filter tended to increase and the distribution tended to skew slightly in the positive direction. For long sequences of censored trials, the accumulated skewness of the exact posterior could become substantial.

In contrast, the posterior mean computed using the data deletion approach does not incorporate the information that censoring is more likely to occur for certain state values, which can lead to substantial bias in the estimates. The facts that at high levels of censoring only extreme values of the observations—and, hence, the state—are observed, and that the state model imposes a probabilistic continuity constraint, can lead to bias in the estimation procedure. The variance of this distribution does increase during missing-data trials, since the state model tends to increase the uncertainty with each step, and observations, which tend to decrease uncertainty, are missing. However, the skewness of the posterior distribution of the data deletion filter will not adjust accurately on censored trials.

On trials with missing data, the posterior mean computed using the data imputation approach does tend to move in the correct direction to increase the probability of a censored trial. This occurs because the imputation procedure requires that the imputed reaction time be above the censoring threshold. The bias of the data imputation filter is therefore decreased relative to the data deletion approach when the number of trials with missing data is small. When there are long sequences of censored trials, error in the state estimates can lead to poor imputed data values, which in turn can exacerbate the estimator error. Another notable source of error from the data imputation approach relates to the higher-order moments of the estimated posterior distribution. The variance of this distribution can be inappropriately low if the imputed data are treated as being as reliable as the observed data. Finally, the data imputation method tends not to reflect the skewness that develops in the exact posterior during long sequences of censored trials.

In our simulation study, the performance of the approximate Gaussian filter was nearly identical to that of the exact filter. As we discussed above, the exact posterior distribution can become markedly skewed during long sequences of censored trials, which would not be captured by the approximate Gaussian filter. This did not lead to major degradation of the RMSE or HPD coverage in our simulations. On the other hand, a major advantage of the approximate Gaussian filter is that it is computationally efficient, especially as the dimension of the state process increases.

Here we developed a set of filter algorithms for right-censored reaction time signals. The proposed methodology can be extended to other missing-data scenarios; to run the filter, we only need to specify the correct likelihood function of the missing data. For instance, in some behavioral tasks there may be a minimum limit on the detectable reaction time, and very quick responses may therefore also be censored. In that case, the filter paradigm would still follow the structure of Equations 5 and 6 but would require a specification of the likelihood that reflects this new censoring process. Appendix A describes the likelihood function of two-sided censored data (Yousefi et al., 2017). We could similarly compute an approximate Gaussian filter based on this likelihood that reflects this censoring process.

Here, we demonstrated an application of an exact, full-likelihood filter—and its Gaussian approximation—for state estimation of a mixed behavioral signal with continuous and binary outputs. There is little established technique for addressing missing-data problem for mixed ob servations; in fact, limited estimation techniques are available for the fully observed case. Both the exact and the Gaussian approximation approaches proposed here can be applied to more complex behavioral signals—for instance, behavioral signals with nonnormal distributions or signals with a mixture of skewed and multinomial distributions (Yousefi et al., 2015). Furthermore, the Gaussian approximation might be a computationally preferable solution for filter problems with a high-dimensional state variable (more than one or two dimensions), for which the computational cost of the exact filter is excessive, and we generally look for an approximate posterior estimate. The Gaussian approximate method is limited by a matrix inversion step, which should scale polynomially in *n*, the dimension of the state process—*O*(*n*^3^) or better; that is, a simple numerical solution to the full-likelihood filter will scale exponentially in *n* (Gordon, Salmond, & Smith, 1993; Masreliez, 1975).

This filter paradigm is not limited in application to behavioral reaction time data. Similar problems of estimating dynamic state processes from partially censored observations processes arise in many other fields, including engineering and medicine. For instance, electrophysiological signals may be lost due to an amplifier’s limited dynamic range, or to temporary saturation from an applied electrical stimulation. Imaging data can include pixels that are saturated or corrupted by a saturating noise. Chemical analytes such as neurotransmitters may reach concentrations above or below a test’s detection range. The approaches proposed here can be extended for such signals, to generate better procedures for estimating the dynamical properties of a system.

Here we have focused on the filter problem of estimating the state process at the current trial using the observations up to and including the current trial. Often when analyzing completed experiments, we are more interested in solving smoothing problems. For example, the fixed-interval smoothing problem attempts to estimate the state process at each trial using all of the observed data over the experiment, including those from later trials. It is easy to show that in the presence of censored data, this smoothing problem can be solved by a two-step procedure, in which the first step involves iterating forward a filter algorithm such as the ones described here, and the second step involves iterating a smoothing expression backward in time. The smoothing step would not be affected by the censoring process. Therefore, for the state model in Equation 8 we could incorporate the smoothing step for a standard Kalman smoother with our censored filter (Särkkä, 2013).

In this work, we proposed methodologies to track the dynamics of an unobserved process that can only be inferred using a set of partially censored observations, assuming that models for the dynamics of the process and for the observations are known. This does not address the problem of identifying those models or estimating their parameters. An important extension of these methods would be to estimate simultaneously some set of the model parameters in Equations 8, 9, and 10 while tracking the state variable(s). Approaches to perform such simultaneous parameter and state estimations include an expectation maximization (EM) algorithm or a variational Bayes (VB) method (Beal, 2003; Bernardo et al., 2003; Smith & Brown, 2003). The EM algorithm is an iterative method for finding maximum-likelihood parameters in statistical models in which the model depends on unobserved variable(s) (Dempster, Laird, & Rubin, 1977). For the dynamic behavioral model proposed here, for instance—the model defined in Equations 8, 9 and 10—the EM algorithm consists of two steps: (1) the E-step, which runs an exact filter and smoother with the current parameter estimates to obtain an update to the state estimates, and (2) the M-step, which uses the filtered and smoothed state estimates in a maximum-likelihood calculation to obtain an update to the parameter estimates—for example, $\left(a_{0},a_{1},b_{0},b_{1},c_{0},c_{1},c_{2},\sigma_{\varepsilon }^{2},\sigma_{v}^{2}\right)$, the parameters in Equations 8, 9, and 10. The exact-filter and smoother algorithms for the E-step rely on fully observed behavioral signals, and the filter and smoother algorithms both need to be modified for signals with missing/censored data. The exact filter—and smoother—proposed here can be utilized in the state-space EM method to provide an accurate estimate of the state process. It is worth mentioning that the algorithm to perform the M-step needs to account for the missing data from censored trials. Future work will focus on developing an EM algorithm for censored data. The VB method is similar to the EM algorithm, except it uses a Bayesian framework and estimation methods to compute the posterior distribution of the model parameters and hyperparameters using the censored filter output. Besides the EM and VB methods, Mohan, Pearl, and Tian (2013) discussed the conditions that are necessary for consistent estimations using data missing not at random in causal graphical models (Mohan et al., 2013; Thoemmes & Mohan, 2015). Van den Broeck, Mohan, Choi, and Pearl (2014) also proposed an alternative parameter estimation method using a deletion-based approach, which outperforms the EM algorithm when the data set has a substantial number of missing data points. Thoemmes and Rose (2014) studied the use of auxiliary variables in missing-data problems and provide recommendations to include variables that avoid detrimental effects and increased bias. Mathys et al. (2014) proposed a hierarchical Gaussian filter to model uncertainty in perception tasks; they also demonstrated applications of different inference algorithms, including VB, to estimate uncertainty quantity and model parameters given observed decisions. Future work will focus on further development of such parameter estimation algorithms for censored data.

Here, we have proposed a general framework to deal with missing data in dynamic behavioral signals. Missing data are a common problem across different research domains in which dynamic processes influence the observed data, and the methodology we proposed here is likely to be applicable to a wide variety of such missing-data problems (Stekhoven & Bühlmann, 2012; Troyanskaya et al., 2001; Vaishnav & Patel, 2015). Moreover, the fact that this algorithm encompasses the data deletion, data imputation, and full-likelihood approaches to missing data makes this approach useful for a wide range of problems. Finally, simulation studies such as the one provided here can provide direct guidance to researchers about which specific approach for dealing with missing data would be most appropriate for any analysis.

## AUTHOR CONTRIBUTIONS

A.Y. - Development of the method, implementation of the algorithm, and writing the manuscript. E.N.S. - Contributed to preparation of the manuscript. D.D.D. - Contributed to preparation of the manuscript. A.S.W. - Designed research, assisted with data collection, edited the manuscript. U.T.E. - Assisted in development of the method, implementation of the algorithm, and writing the manuscript.

## ACKNOWLEDGMENTS

This research was funded in part by the Defense Advanced Research Projects Agency (DARPA) under Cooperative Agreement Number W911NF-14-2-0045, issued by the Army Research Office contracting office in support of DARPA’s SUBNETS program. The views, opinions, and/or findings expressed are those of the authors and should not be interpreted as representing the official views or policies of the Department of Defense or the U.S. Government.
